# Country-by-Country Reporting: A Step Towards Unitary Taxation?

**DOI:** 10.1007/s10272-021-0974-9

**Published:** 2021-06-04

**Authors:** Miguel Viegas, António Dias

**Affiliations:** 1 GOVCOPP, DEGEI, Universidade of Aveiro, Campus Universitário de Santiago, 3810-193 Aveiro, Portugal; 2 CETRAD, Universidade of Trás-os-Montes e Alto Douro, Quinta de Prados, 5000-801 Vila Real, Portugal

## Abstract

Multinational companies are now obliged to deliver an annual report to the tax authorities with information disaggregated by country (country-by-country reporting) in order to show where the assets and workers are allocated, how profits are distributed and to whom taxes are paid. Unfortunately, these reports are not made public in the European Union, thus preventing public scrutiny about the strategies used by multinational companies to displace profits to tax havens. This article applies the Unitary Taxation regime proposed by the European Commission to US multinational companies. The results confirm a strong bias among the profits distribution towards countries with lower corporate tax rates. Likewise, they confirm the capacity of the Unitary Taxation to promote a fairer distribution of tax revenues. These results can be a good contribution to the current Portuguese presidency of the European Union, which managed to gather important support to move forward with the European public country-by-country reporting directive.

According to the European Commission's latest summer forecast, the economic recession caused by the COVID-19 crisis will be even worse than predicted in previous reports. In 2020, the EU economy experienced a deep recession despite the rapid and comprehensive response from the EU and each of its member states. In addition to the effects on the economy, the budgetary consequences will be equally severe. Not by chance, the European Commission proposed, and the Council accepted, the activation of the general escape clause of the Stability and Growth Pact. This clause will provide member states with greater budgetary latitude to better respond to the ongoing economic and social crisis. This clause was introduced in 2011, in the wake of the 2008 financial crisis. However, as stated by the European Commission, the activation of this general escape clause cannot jeopardise the sustainability of public finances. It only allows a temporary deviation from the medium-term objectives to deal with serious events that governments cannot control and that have a strong impact on public finances.

**Miguel Viegas**, University of Aveiro, Portugal.

**António Dias**, University of Trás-os-Montes and Alto Douro, Portugal.

Therefore, the need to find budgetary resources to boost the economy and balance public finances is now more prominently placed on the agenda than ever before. According to conservative estimations from the European Union, revenue losses due to corporate tax avoidance in the EU could amount to around €160-€190 billion annually.[1] In addition to this breach in public accounts, tax fraud and tax evasion distort competition in favour of large multinational companies, whose tax bill is 30% lower than that of small and medium-sized enterprises. Quoting the figures from the European Commission, a fairer tax collection would allow for the easing of income of 80% of families. In light of the current crisis, but also keeping in mind the challenges that remain on the climate agenda, combating corporate tax evasion and tax avoidance should be a top priority.

The fight against fraud and tax evasion within the European Union was greatly boosted by the Luxleaks scandal in 2014. This investigation revealed the tax rulings set up between Luxembourg's government and over 300 multinational companies and strongly affected the credibility of the European Commission and its President, Jean-Claude Juncker.[2] The most important measures against fraud and

1 https://www.europarl.europa.eu/legislative-train/theme-deeper-and-fairer-internal-market-with-a-strengthened-industrial-base-taxation/file-quantification-of-the-scale-of-tax-evasion-and-avoidance.

2 Jean-Claude Juncker was Minister of Finance and Prime Minister of Luxembourg for 21 years.

tax evasion were guided along three main axes: improving international cooperation, increasing transparency and combatting aggressive tax planning.[3] This article explores two comprehensive measures: country-by-country reporting and the creation of a unitary taxation framework.

Country-by-country reports (CbCR) were incorporated into European legislation in 2016. Since then, it has been revised with the intent of increasing transparency. However, a European agreement for its public disclosure has not yet been possible. The public disclosure is necessary to allow real scrutiny of how multinational companies distribute their assets across nations, where they carry out their activity, declare their profits and pay their taxes. Unlike the European Union, the CbCR of US multinational companies is publicly disclosed by US tax authorities. The aggregated and properly treated information from the CbCR has been available on the Internal Revenue Service's (IRS) website since 2016.[4]

In this article, we use this database containing information on 1,205 multinationals headquartered in the US and with branches in the EU. The data includes information on the activity of the companies for 2016 and 2017. The delivery of CbCRs by multinational companies started in 2016 and these are, so far, the only data available. Crossing profits with indicators of physical presence of these companies, we conclude that there is a clear concentration of the taxable profits in countries with lower corporate tax rates. The application of a unitary taxation framework results in a substantial redistribution of declared profits from the fiscally attractive countries to the countries where the multinationals operate. Therefore, a new unitary taxation regime would contribute to OECD objectives and its Base Erosion and Profit Shifting (BEPS) project, ensuring that profits are taxed where economic activities are performed and where added value is created.[5] At the same time, it would give member states more tax revenue to help relaunch their economies and avoid over-penalising household disposable income.

## The EU's proposals for country-by-country reporting and the conception of a unitary taxation

The CbCR were created to strengthen the cooperation between tax administrations. Directive 2011/16/EU, known as the Directive of Administrative Cooperation (DAC 1) established the legal basis for the administrative cooperation in the field of taxation within the European Union. This directive has been revised several times:

- Directive 2014/107/EU (DAC 2) introduces the automatic exchange of information;[6]
- Directive 2015/2376/EU (DAC 3) includes transfer pricing agreements in the automatic exchange of information;
- Directive 2016/881/EU (DAC 4) includes the CbCR in the automatic exchange of information;
- Directive 2016/2258/EU (DAC 5) provides access to information on anti-money laundering measures;
- Directive 2018/822/EU (DAC 6) includes tax rulings in the automatic exchange of information.

The directive was later revised to broaden the field of information included in these automatic exchanges of information. The CbCR were formally created in the third review (DAC 4).

According to Directive 2016/88, all companies with total consolidated group revenue of at least €750 million are required to report detailed country-by-country information on revenues, profits, taxes paid, capital, earnings, tangible assets and the number of employees. Any multinational company – European or not – that is currently active in the EU's Single Market with a permanent presence must comply with these additional transparency requirements. The directive implemented Action 13 from the OECD BEPS project.[7] The CbCR must be included in the automatic exchange of information between all member states where the multinational company has reported activity. However, CbCR information cannot be disclosed to the public as was initially proposed by the European Commission.

CbCR were then seen only as a tool by the tax authorities to infer eventual abuses of the transfer pricing system with the purpose of draining profits into tax havens. Their scrutiny by political representatives, journalists, NGOs and the academic community was never considered socially relevant. However, after the successive fiscal scandals that followed Luxleaks, the atmosphere changed. Public opinion began to demand greater transparency in the way multinationals are taxed. The proposal to make the CbCR public re-entered the European Commission's agenda, and Portugal's EU Presidency recently achieved a broad consensus among EU countries to move the proposal forward.

In 2016, the European Commission re-launched a proposal made in 2011 to create a unitary taxation for multinational companies within the EU. The Commission proposal (Common Consolidated Corporate Tax Base, CCCTB) was presented as the most ambitious corporate tax reform

3 For a review of the fight against fraud and tax evasion in the European Union since Luxleaks, see Viegas (2018).
4 The IRS is the agency of the United States Treasury Department responsible for tax collection.
5 https://www.oecd.org/tax/beps/beps-actions/.
6 The exchange of information between governments normally proceeds in three different ways: exchange of information upon request, spontaneous exchange of information and automatic exchange of information.
7 https://www.oecd.org/tax/beps/beps-actions/action13/.

Box 1
**Unitary taxation**

There is nothing new about the concept of unitary taxation. The United States has extensive experience with this type of taxation going back to the 1930s when the California state government prevented large companies linked to film production from draining their profits through branches located in the state of Nevada where taxation was lower (Wiederstein, 1992; Picciotto, 2012). Unitary taxation was also applied to the extractive industry (Agostini, 1988). This application was always very controversial and was never imposed outside the US due to strong opposition from big multinational companies and their powerful lobbies. Thus, what prevailed as the internationally accepted standard was the so-called arm's length principle and the separate entity, according to which every entity (including each enterprise within the multinational enterprise group) is considered a separate and independent taxpayer. Moreover, a transaction respects the arm's length principle when both parts act as if they are completely independent with no relationship to each other. The concept is used to ensure both parties in the deal are acting in their own interest and are not subject to any pressure from the other party. In other words, the branch separation of multinational companies is accepted, but the principle of free and fair competition is applied to intra-group transactions to avoid artificial profit relocations for tax savings purposes. The prices practiced in such intra-group transactions must not deviate from the value practiced in the free market between independent companies.

The arm's length principle and the separate entity approach adopted by OECD member countries are, in some cases, difficult to apply. Associated enterprises frequently engage in transactions with no comparable terms in free markets, such as those dealing with highly specialised goods, unique intangible assets or specialised services.

The application of a unitary taxation must be based on three fundamental principles (Picciotto, 2012). First, it must establish the perimeter of the multinational company subject to taxation. This implies the determination of criteria that establishes the degree of ownership of the assets and the chains of control and subordination of the various entities in different countries. Second, common accounting rules should be established for calculating the tax base. Third, a formula must be established to distribute the taxable profit across the various tax jurisdictions where the multinational company operates. The most common elements of weighting are sales, number of workers (or labour costs) and tangible assets.

The unitary taxation allows for the overcoming of the great difficulties of application of the arm's length principle. Its saves tax authorities' resources and ensures that profits are actually taxed where they are created. It also has the advantage of promoting international accounting harmonisation, easing international trade and investment (McGaughey and Raimondos, 2019).

ever proposed in the EU (see Box 1 for an account of a unitary taxation approach).[8] The CCCTB will be mandatory for all groups with global consolidated revenues of more than €750 million. The new proposal is split into two proposals which can be implemented in two stages. Firstly, member states must agree on the common corporate tax base (CCTB). The CCTB proposal provides the single set of rules to calculate the company's tax base with the harmonisation of various exemption and deduction regimes. This means that companies will only have to refer to one set of rules when calculating their taxable profits and the calculation will be uniform throughout the EU. These common rules for taxing companies in the EU would remove the loopholes and mismatches in the current corporate tax frameworks that enable aggressive tax planning. These new rules support research and development (R&D) deduction and remove the incentive for debt accumulation (through interest deduction limits). Secondly and after the tax base has been established, the company's consolidated taxable profits are split between the member states in which the company is active using an apportionment formula. This formula is based on three equally weighted factors: the assets, labour and sales that the company has in each member state. The member states can then tax their share of the company's profits applying their own national rate. According to Morgan (2016) the creation of a unitary taxation represents the only way to face the new segmentation of multinational companies and guarantee the principle according to which profits should be taxed where they are generated.

The CCCTB proposal to create a unitary tax in the EU imposed on multinationals could benefit from a new impetus in the context of the ongoing discussion on how to pay the €750 billion loan made on behalf of the EU to finance

8 https://ec.europa.eu/taxation_customs/business/company-tax/common-consolidated-corporate-tax-base-ccctb_en.

Figure 1
**Multinationals per member state**

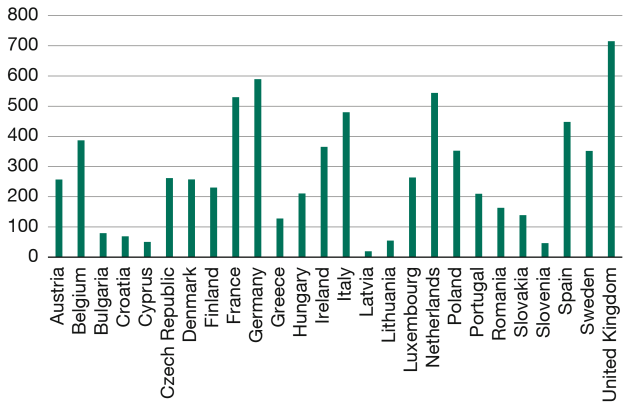

Source: Internal Revenue Service (2019).

Figure 2
**Multinationals per million inhabitants and corporate tax rate**

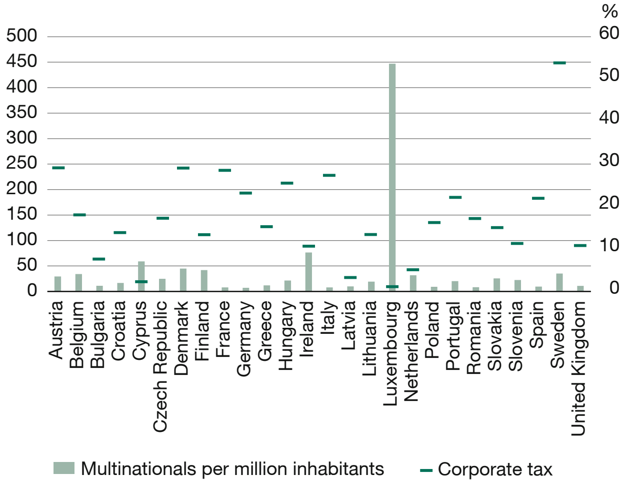

Source: Internal Revenue Service (2019); and authors' calculations.

the Next Generation EU recovery fund. New resources are needed to prevent member states from being called upon to repay the loan from 2028 onwards. The European Commission has put several proposals on the table to feed the EU budget. The list of possible new revenues includes a common consolidated corporate tax base, taxes on digital services, a financial transaction tax, taxes on the EU Emissions Trading Scheme, a levy on plastics and on products imported to the EU that were produced under lower $CO_2$ emissions standards than in the EU. This new tax on large multinational companies with a turnover above €750 million would be obtained from a fraction (3%) of the consolidated common tax base resulting from the application of the European Commission's CCCTB proposal. According to the European Court of Auditors' (2018) calculations, this proposal would represent an annual revenue of €12 billion.

## Country-by-country reports of US multinationals

In the US, the CbCR is mandatory for multinational enterprises with more than $850 million of revenue since 2016. The report must include sales, assets, taxes paid and other indicators of economic activity on a country-by-country basis. The data are communicated to the Internal Revenue Service using a specific form. The information is subsequently processed by the IRS and publicised through its internet portal. The database used in our exercise contains information on 8,975 multinational enterprise residents in the US and refers to the period 2016-2017. The information, disaggregated by countries, covers sales volume, taxable profits (or losses), taxes paid, the number of workers and fixed assets.

We start by analysing the location of multinationals within the European Union. Figure 1 illustrates the number of multinationals present in each member state. The two countries with the largest presence of multinational companies in their territory are the United Kingdom and Germany. However, if we look at Ireland, the Netherlands and Luxembourg, we observe a disproportionate number of multinationals present for the size of these countries, whether in terms of physical, economic or demographic dimension. If we calculate the number of multinational companies per million inhabitants, we see that this ratio is seven in Germany, eight in France, 32 in the Netherlands, 77 in Ireland and 447 in Luxembourg. It seems clear that there is a strong attraction to multinational companies for specific countries.

Figure 2 introduces a first clue by comparing the real income tax rate on profits with the ratio of multinational companies per million inhabitants. The curve of the corporate tax rate shows a pattern inversely proportional to the intensity of the presence of multinational companies. Looking left to right, we identified a first break with Cyprus, which has 59 multinationals per million inhabitants and a real tax rate on profits of 1.76%. The second case is Ireland with 77 multinational companies per million inhabitants and a real tax rate of 10.92%. The third case corresponds to Luxembourg and the Netherlands where the tax rates are 0.92% and 5.16%, respectively. Finally, we have the United Kingdom, the country with the largest presence of multinational companies in absolute values (716 multinational enterprises operating in the UK although only 11 per million inhabitants) and with a real tax rate of 10.35%. The average corporate tax rate in the sample is 21.30%. These results clearly indicate a pref-

Figure 3
**Productivity of multinational companies per worker**

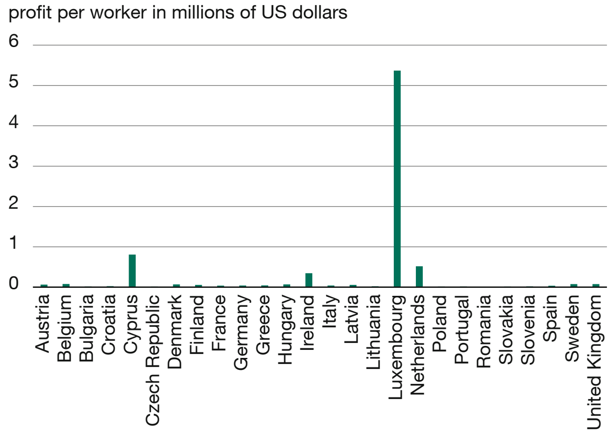

Source: Internal Revenue Service (2019); and authors' calculations.

erential location for multinational companies in countries where the fiscal framework is more favourable.

Another way of showing the location bias of multinational companies towards most favourable tax jurisdictions is to study the distribution of profits before tax. The so-called profit shifting is a well-known practice used by multinationals to drain taxable income towards jurisdictions with low or even zero tax rates. Through their numerous subsidiaries or shell-companies, multinational companies use internal transactions to claim expenses and losses in high-tax countries while profits are declared in low-tax jurisdictions. Today, more than two-thirds of world trade takes place within multinational enterprises (Garbarino, 2012). Transfer pricing manipulation, used by multinational companies to shift profits from places where they are produced towards low-tax territories, has received significant attention from the OECD. In the BEPS plan, several actions are dedicated to preventing and combatting abusive practices in terms of transfer prices (Actions 8, 9 and 10).

Figure 3 illustrates the productivity (profit) of multinational companies per worker in each member state. Once again, the chart identifies a small group of countries where this productivity is significantly higher relative to other countries. Luxembourg, Ireland, the Netherlands and Cyprus are among the most representative examples. According to the figures, we could conclude that a worker in Luxembourg produces 312 times more than a Portuguese worker on a daily basis. Similarly, a Cypriot, Dutch or Irish worker would produce, respectively, 47, 30 and 20 times what a Portuguese worker produces in the same period. Obviously, these disparities do not reveal any incapacity for Portuguese

Table 1
**Tax base redistribution across EU members with unitary taxation, in millions of US dollars**

| Country | Profit before tax | Profit with unitary tax | Gain/loss |
|---|---|---|---|
| Austria | 1,404.14 | 1,983.24 | 579.10 |
| Belgium | 6,623.22 | 12,944.01 | 6,320.80 |
| Bulgaria | 234.98 | 864.20 | 629.23 |
| Croatia | 111.23 | 215.39 | 104.17 |
| Cyprus | 1,112.48 | 105.19 | -1,007.29 |
| Czech Republic | 1,172.13 | 4,007.33 | 2,835.20 |
| Denmark | 1,584.92 | 1,958.35 | 373.43 |
| Finland | 958.29 | 1,609.83 | 651.54 |
| France | 9,140.92 | 19,544.58 | 10,403.66 |
| Germany | 17,022.11 | 32,860.18 | 15,838.07 |
| Greece | 449.73 | 644.65 | 194.92 |
| Hungary | 4,816.56 | 3,764.72 | -1,051.84 |
| Ireland | 39,571.39 | 26,328.82 | -13,242.58 |
| Italy | 6,029.04 | 12,191.13 | 6,162.09 |
| Latvia | 34.31 | 31.94 | -2.37 |
| Lithuania | 172.67 | 327.57 | 154.90 |
| Luxembourg | 41,879.60 | 21,537.69 | -20,341.91 |
| Netherlands | 57,771.97 | 22,430.86 | -35,341.11 |
| Poland | 2,707.22 | 9,872.79 | 7,165.57 |
| Portugal | 566.62 | 1,699.84 | 1,133.22 |
| Romania | 561.76 | 2,710.28 | 2,148.52 |
| Slovakia | 536.42 | 1,913.98 | 1,377.56 |
| Slovenia | 74.22 | 217.24 | 143.02 |
| Spain | 5,021.42 | 10,338.54 | 5,317.12 |
| Sweden | 3,024.96 | 3,353.82 | 328.86 |
| United Kingdom | 65,916.09 | 75,042.21 | 9,126.12 |

Source: Authors' calculations based on Internal Revenue Service (2019).

workers or any miracle in the capacity of workers in the other four countries. These disparities simply reveal the existence of aggressive tax planning schemes used to divert taxable profits from less fiscally attractive countries to territories where taxation is low or even zero. This evidence of profit shifting is well described in Garcia-Bernando et al. (2021).

## The opportunity to create a unitary taxation regime

Table 1 exhibits the outcome of the Commission's CCCTB proposal applied to the IRS database. The apportionment formula is applied to the 1,205 US multinationals operating in EU countries. In the first column, we see the pre-tax profits reported by multinational companies in each of the member states (including the United Kingdom). Estonia and

Malta are not on the list as none of the multinationals under study registered any physical presence in these two member states. In the second column, we can see the simulation of what the tax base distribution would be, as proposed by the Commission, with the apportionment formula based on sales volume, number of workers and fixed assets. In the third column, we can see the taxable base gains or losses resulting from the application of the apportionment formula.

Unsurprisingly, the results confirm the existence of a small group of member states that have the power to concentrate taxable profits using a political framework based on fiscal extortion. Applying the allocation formula proposed by the European Commission, six countries are worse off. Leading the countries with the greatest losses is the Netherlands with a shortfall of $35 billion taxable profits, followed by Luxembourg, Ireland, Hungary, Cyprus and Latvia. This new apportionment represents a zero-sum game between EU countries. Therefore, 22 countries will be better off with this new tax base distribution. Among the winners we have Germany in first place, earning almost $16 billion of taxable profits, followed by France, the United Kingdom, Poland, Belgium and Italy. Overall, there are almost €71 billion of taxable profits that return to the countries where they were generated. The dispersion of the tax base distribution, measured by the standard deviation, falls by 13%, thus indicating a more equitable distribution.

Empirical exercises using unitary taxation within the European Union are not abundant in the literature. In an excellent article, Cobham and Loretz (2015) test several formulas of profit redistribution using the Orbis database, provided by the Bureau van Dijk.[9] It is not possible to compare results since the authors use different apportionment formulas. The criteria are the same: assets (total and intangible), sales and workers. However, the weighting is different. The distinction between total assets and fixed assets is useful. It allows highlighting the use of intangible assets as a strategic weapon to relocate profits artificially towards tax havens. Considering the different formulas used in Cobham and Loretz (2015), we find that in all the results, Luxembourg and the Netherlands systematically appear as the countries most penalised by applying the unitary taxation principle. Losses of taxable profits for those countries range from 40% to 80%. These results exhibit the same magnitude as those obtained in our exercise. In the case of Luxembourg and Ireland, according to our calculations, the decrease rates of taxable income caused by the apportionment formula proposed by the European Commission are 48.6% and 61.2%, respectively.

The convergence of corporate tax rates between member states remains a priority for many specialists and authorities. This convergence is necessary to stop the current race to the bottom caused by fiscal competition. However, considering the unanimity rule prevailing in the community decision-making process in tax affairs, this proposal is today considered to be unattainable by the European Commission.[10] When the Commission launched its new proposal to create a tax system based on unitary taxation in 2016, the harmonisation of corporate tax rates was intentionally left out of the proposal because the necessary consensus for its approval had not yet been reached. However, the proposal assures the principle according to which profits should be taxed where they are generated. After a fairer distribution of the tax base, it is up to each member state to apply the tax rate in force in their country. Applying the apportionment formula to the tax base of US multinational companies, we recorded a total redistribution of €71 billion of taxable profit. Applying the average corporate tax rate in force within the European Union (21.30%), we obtain a tax revenue slightly above €15 billion per year, to be distributed among the 22 member states where multinationals are located and operate. This figure gives us a dimension of the ongoing fiscal injustice generated by the current status quo and the benefit that could arise for many EU countries with a new unitary taxation regime.

## Conclusion

Current corporate tax systems and institutions for international cooperation in tax matters were created in the early 20th century. Since then, the process of economic globalisation has completely changed the production and marketing chains. The companies have gained scale and internationalised their business. Taking advantage of digitalisation, companies have branched out, dividing themselves into multiple distinct entities spread across several continents. Today, multinational companies have become the main locomotive of international trade and investment. The current tax system has proved to be unfit for this new reality and unable to promote fair and efficient corporate taxation (Ting and Gray, 2019). The application of the paradigm of separate entities and the arm's length principle has resulted in a complex set of rules that are difficult to apply and has cast doubt on their effectiveness.

The obligation of large multinational companies to disclose their results disaggregated by country is an important step towards more transparency. Contrary to what happens in the European Union, these CbCR are publicly disclosed in the US, thereby allowing for scrutiny of how

9 https://orbis.bvdinfo.com/.

10 For a good review about this issue, consult Bettendorf et al. (2010).

US multinational companies allocate their assets, their workers, where they declare their profits and where they pay their taxes. The IRS database referring to the CbCR of 1,205 US companies confirms the existence of aggressive tax planning schemes prescribed by the biggest accounting firms and used to direct profits artificially towards more tax-attractive member states.

This result, affecting only multinational companies based in the US, underlines once again the injustices of the current tax system and emphasises the opportunity to create a unitary taxation regime. Applying the apportionment formula proposed by the European Commission in 2016 results in a substantial redistribution of the tax base across the EU member states (including the United Kingdom). As expected, this redistribution strongly penalises the "usual suspects": the Netherlands, Luxembourg and Ireland, among others. Overall, €71 billion of taxable profit returns to the countries where it was generated.

The unitary taxation regime, which considers the multinational company as a whole, has several advantages over the current dominant paradigm of separate entities and the principle of full competition (Picciotto, 2012). With a unitary taxation regime, it is not necessary to know all the details of the companies' internal accounting and the respective transfer pricing system applied to intra-group exchanges. The complex anti-abuse laws to combat aggressive tax planning schemes (interest deductions, R&D expenses, etc.) cease to be the principal focus of tax administrations. Finally, disputes over the recognition of the status of permanent establishment are also less important.

This tax reform is controversial within the European Union. This article offers a concrete application of a unitary tax on a small sample of multinational companies and allows us to better understand the opposition of several member states that are, today, the main beneficiaries of the status quo. Public disclosure of CbCR could contribute to breaking the current impasse in the Council. In the last few years, several tax scandals have shaken public opinion (Luxleaks, Swiss Leaks, Panama Papers, etc.), revealing the tax impunity of multinational companies in contrast to SMEs and most European citizens. Following the Panama Papers, the European Commission proposed a directive to impose a CbCR for big multinational companies from all branches of activity, with public disclosures of the information. This proposal is not without criticism. It only covers companies with a turnover of more than €750 million. It only requires information about activities within the European Union and in the countries included in the so-called third-country black list, which covers about a dozen countries. Therefore, this proposal leaves out a significant part of multinationals that sell less than €750 million. Moreover, it gives the possibility for multinational companies covered by the Directive to export profits to a wide range of third countries not covered by the black list.

This proposal has been blocked in the Council. Recently, the current Portuguese presidency of the European Union obtained the support of the main member states to unblock the situation. However, its approval implies unanimity, and therefore, it is not yet guaranteed. Despite the criticism of the proposal, its approval and application would expose to civil society the unfair consequences of aggressive tax planning schemes of multinational companies. Moreover, it would also expose the role of certain member states that encourage these schemes embedded in a model of tax predation. This public scrutiny, coupled with the growing need for public resources required to face the current economic crisis caused by the COVID-19 crisis, could be the catalyst for a comprehensive reform of the international tax system with the implementation of a unitary taxation regime for multinational companies. We hope that this article will be a contribution in this direction.
